# Case Report: Neoadjuvant chemoimmunotherapy achieving pathological complete response in two cases of stage III EGFR-mutant NSCLC with high PD-L1 expression

**DOI:** 10.3389/fonc.2026.1844982

**Published:** 2026-06-17

**Authors:** Diming Wang, Tangbin Liu, Jian Zhao, Bowen Ding, Yongliang Niu

**Affiliations:** 1 Department of Oncology, Anhui Chest Hospital, Hefei, China; 2 Department of Thoracic Surgery, Anhui Chest Hospital, Hefei, China; 3 Department of Pathology, Shanghai Chest Hospital, Shanghai Jiao Tong University School of Medicine, Shanghai, China; 4 Department of Respiratory and Critical Care Medicine, No.2 People’s Hospital of Fuyang City, Fuyang, China

**Keywords:** EGFR-mutant, lung adenocarcinoma, neoadjuvant chemoimmunotherapy, neoadjuvant immunotherapy, NSCLC

## Abstract

Resectable locally advanced EGFR-mutant non-small cell lung cancer (NSCLC) represents a distinct therapeutic challenge. Owing to an inherently immunosuppressive tumor microenvironment—frequently characterized by absence of or low PD-L1 expression and sparse CD8+ tumor-infiltrating lymphocytes—patients with EGFR alterations have been routinely excluded from landmark perioperative immunotherapy trials or have demonstrated negligible pathological responses. Consequently, the optimal neoadjuvant strategy for this population remains undefined, and the potential efficacy of immune checkpoint inhibitors combined with chemotherapy in selected EGFR-mutant subtypes is largely unexplored. We report two patients with resectable stage IIIA lung adenocarcinoma and concurrent high PD-L1 expression (tumor proportion score 60%) who achieved pathological complete response (pCR) following neoadjuvant chemoimmunotherapy. Case 1, a 60-year-old male harboring an EGFR exon 19 deletion, received three cycles of pemetrexed, carboplatin, and pembrolizumab, followed by R0 resection; postoperative adjuvant pembrolizumab was discontinued after one cycle because of grade 2 dizziness (CTCAE v5.0). Case 2, a 52-year-old female with an EGFR exon 21 L861Q missense mutation co-occurring with TP53 and PIK3CA alterations, exhibited primary resistance to afatinib with radiographic progression after one month. She subsequently underwent three cycles of pemetrexed, carboplatin, and sintilimab, achieving R0 resection with pCR, and declined further postoperative therapy. After 16 and 22 months of follow-up, respectively, the two patients remain disease-free. These cases suggest that for a specific subset of EGFR-mutant NSCLC—particularly those with high PD-L1 expression and in whom targeted therapy has failed—neoadjuvant chemoimmunotherapy may serve as a potent individualized strategy capable of overcoming pre-existing immune tolerance and inducing profound pathological responses. Nevertheless, this evidence remains strictly anecdotal; validation through large-scale, prospective, biomarker-driven clinical trials is imperative before any modification of standard clinical practice.

## 1 Introduction

Currently, EGFR(Epidermal Growth Factor Receptor) tyrosine kinase inhibitors (TKIs) represent the preferred first-line treatment for advanced EGFR-mutant lung adenocarcinoma. However, the optimal neoadjuvant strategy for patients with resectable locally advanced EGFR-mutant lung adenocarcinoma remains undefined. In the phase III NeoADAURA trial (1), neoadjuvant osimertinib, either with or without chemotherapy, resulted in major pathological response (MPR) rates of 26% and 25%, respectively, in patients with resectable stage II–IIIB EGFR-mutated NSCLC—significantly higher than the 2% rate observed with chemotherapy alone. The corresponding pathological complete response (pCR) rates were 4% and 9%, compared to 0% in the control group. Nevertheless, these pCR rates remain suboptimal.

In contrast, adjuvant immunotherapy has markedly improved outcomes in driver gene-negative populations. Multiple studies have established neoadjuvant immunotherapy combined with chemotherapy as an effective approach for stage IIIA disease, demonstrating superior pathological responses and reduced recurrence risk (2, 3). These trials, however, generally excluded patients with EGFR or ALK alterations, as such tumors are known to respond poorly to PD-1/PD-L1 inhibition.

However, this conventional perception has been increasingly challenged by emerging prospective evidence. The phase 2 CTONG2104/NEOTIDE trial (NCT05244213) evaluated neoadjuvant sintilimab combined with carboplatin and nab-paclitaxel in 18 patients with resectable EGFR-mutant NSCLC (4). All patients completed three cycles of neoadjuvant therapy and proceeded to radical resection without intra-induction disease progression. The study reported a major pathological response (MPR) rate of 44.4% (8/18), with four cases achieving near-pCR (residual viable tumor ≤1%); however, no true pathological complete response (pCR) was observed. The regimen exhibited acceptable clinical feasibility and a manageable safety profile. Mechanistically, platinum-based chemotherapy can induce immunogenic cell death and promote dendritic cell maturation through damage-associated molecular patterns, thereby enhancing tumor immunogenicity and T-cell activation. Concurrently, cytotoxic agents may attenuate immunosuppressive populations such as regulatory T cells and myeloid-derived suppressor cells, potentially converting an immune-”cold” microenvironment into one susceptible to PD-1 blockade (5). Complementary data from neoadjuvant atezolizumab plus chemotherapy also produced pCR in 2 of 4 EGFR-mutant patients (6). Collectively, these findings suggest that neoadjuvant chemoimmunotherapy possesses measurable activity in selected EGFR-mutant NSCLC, yet confirmed pCR remains unreported in prospective cohorts, and the therapeutic potential of the high–PD-L1–expressing subset remains undefined.

Consequently, research on neoadjuvant chemoimmunotherapy for resectable advanced EGFR-mutant lung adenocarcinoma remains scarce. This gap is largely attributable to the conventional understanding that immunotherapy exhibits limited efficacy in EGFR-driven tumors, a phenomenon frequently associated with an immune-”cold” phenotype characterized by a tumor microenvironment deficient in concurrent PD-L1 expression and CD8+ tumor-infiltrating lymphocytes (TILs) (7). As a result, targeted therapy continues to be the mainstay for this patient subgroup. However, whether EGFR-mutant patients with high PD-L1 expression can derive benefit from neoadjuvant immune checkpoint inhibitors combined with chemotherapy is still an open question. Herein, we report two such patients who achieved a pathological complete response (pCR) following this regimen administered as neoadjuvant therapy.

## 2 Case presentation

### 2.1 Case 1

**Figure 1:**
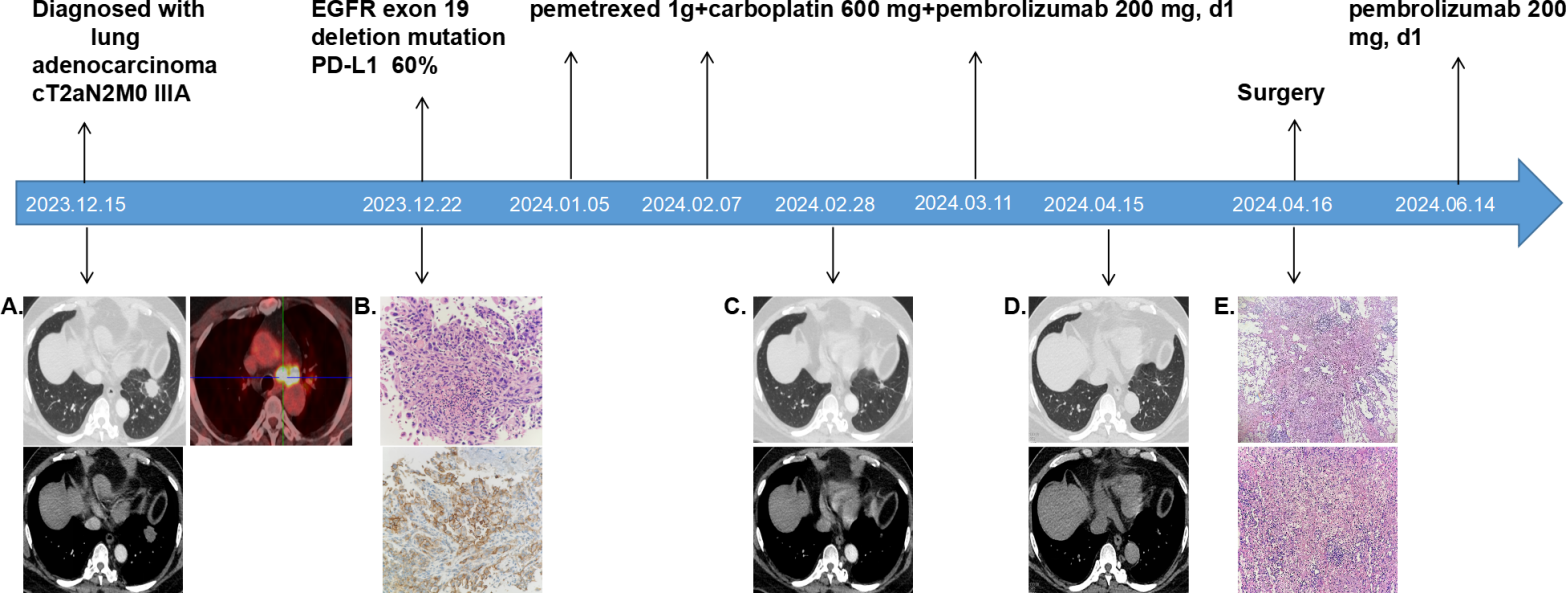
Treatment timeline during neoadjuvant therapy and subsequent surgery. **(A)** Positron emission tomography/computed tomography (PET/CT) scan reveals a mass in the left lower lobe, along with an enlarged mediastinal lymph node and FDG uptake in the 4L lymph node. **(B)** Next-generation sequencing (NGS) identifies an EGFR exon 19 deletion mutation. Lung biopsy pathology (H&Estain,40×) shows tumor morphology. Immunohistochemical analysis shows PD-L1 expression (22C3 antibody, TPS 60%). **(C)** CT images after two cycles of neoadjuvant therapy demonstrate a reduction in the size of the tumor lesion. **(D)** CT images after three cycles of neoadjuvant therapy show a continued reduction in tumor size. **(E)** Postoperative pathology shows no residual tumor at 20× and 40× magnification, indicating a pathologic complete response (pCR).

A 60-year-old Asian male, with a history of hypertension, diabetes mellitus, and bronchial asthma and no smoking history, presented with a left lung mass identified on chest computed tomography (CT) performed on December 14, 2023. His ECOG performance status was 1. A subsequent positron emission tomography-computed tomography (PET-CT) scan on December 15, 2023, revealed a 3.4 × 2.9 cm (**Figure 1A**) soft tissue lesion in the left lower lobe with lobulated and spiculated margins and significantly increased tracer uptake (maximum standardized uptake value [SUVmax] of 25.8). Multiple enlarged lymph nodes were observed in the left hilum and mediastinum (stations 2L, 3A, 4L, and 7), with the largest node measuring approximately 3.0 cm in short diameter and demonstrating markedly elevated 18F-fluorodeoxyglucose (FDG) uptake (SUVmax approximately 22.1), suggestive of metastatic disease. A CT-guided lung biopsy was performed on December 22, 2023. Pathological examination indicated poorly differentiated non-small cell carcinoma, not otherwise specified. Immunohistochemistry (IHC) results were as follows: CD56(-), P40(-), NapsinA(+), CK(+), TTF-1(+), and KI-67(30%+). Molecular profiling by next-generation sequencing (NGS) detected an EGFR exon 19 deletion mutation (19del). PD-L1 expression was 60% on tumor cells (22C3 antibody, DAKO platform) (**Figure 1B**). The final diagnosis was left lung adenocarcinoma, clinical stage cT2aN2M0 IIIA (American Joint Committee on Cancer [AJCC] 8th edition). The patient received two cycles of first-line chemoimmunotherapy on January 5 and February 7, 2024, consisting of pemetrexed 1 g + carboplatin 600 mg + pembrolizumab 200 mg, d1. A follow-up chest CT on February 28, 2024 (**Figure 1C**), indicated a partial response (PR). An additional cycle of the same regimen was administered on March 11, 2024. Repeat imaging on April 15, 2024 (**Figure 1D**), confirmed a sustained PR. The patient tolerated three cycles of neoadjuvant chemoimmunotherapy without significant adverse events.

On April 16, 2024, the patient underwent video-assisted thoracoscopic surgery (VATS) left lower lobectomy with systematic lymph node dissection under general anesthesia, achieving R0 resection. Pathological evaluation of both the lung lesion and dissected lymph nodes showed no residual tumor, confirming a pathological complete response (pCR) (**Figure 1E**). The patient received one cycle of adjuvant pembrolizumab 200 mg on June 14, 2024; however, he developed grade 2 dizziness (CTCAE v5.0). He subsequently refused further adjuvant immunotherapy. No further treatment was given thereafter. A follow-up chest CT scan performed on September 5, 2025 (over 16 months post-surgery), demonstrated stable disease with no evidence of recurrence.

### 2.2 Case 2

**Figure 2:**
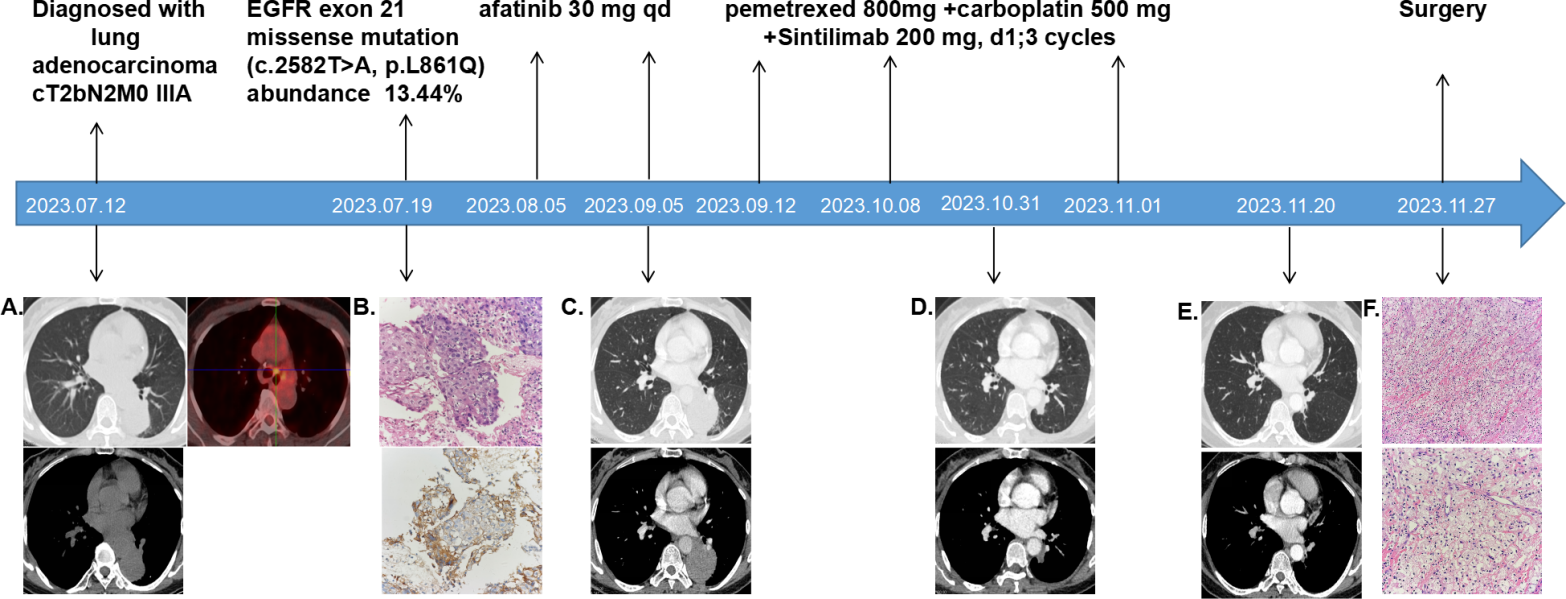
Treatment timeline during neoadjuvant therapy and subsequent surgery. **(A)** Positron emission tomography/computed tomography (PET/CT) scan reveals a mass in the left lower lobe, along with an enlarged mediastinal lymph node and FDG uptake in the 4L lymph node. **(B)** Next-generation sequencing (NGS) identifies an EGFR exon 21 missense mutation (c.2582T>A, p.L861Q) with an abundance of 13.44%. Lung biopsy pathology (H&E stain, 40×) shows tumor morphology. Immunohistochemical analysis shows PD-L1 expression (22C3 antibody, TPS 60%). **(C)** CT images after one month of afatinib (30 mg) therapy demonstrate an increase in the size of the tumor lesion. **(D)** CT images after two cycles of neoadjuvant therapy (pemetrexed 800 mg+carboplatin 500 mg + Sintilimab 200 mg, day 1) show a reduction in tumor size. **(E)** CT images after three cycles of neoadjuvant therapy (pemetrexed 800 mg+Sintilimab 200 mg, day 1) show a further reduction in tumor size. **(F)** Postoperative pathology shows no residual tumor at 20× and 40× magnification, indicating a pathologic complete response (pCR).

A 52-year-old Asian female, with a history of hypertension and prior cholecystectomy for gallbladder stones and no smoking history, underwent a chest CT on July 10, 2023. Her ECOG performance status was 1. The scan revealed a mass in the left lower lobe, mediastinal lymph node enlargement, and multiple solid nodules in both lungs. A PET-CT scan on July 12, 2023, showed a 4.0×3.8×4.3 cm (**Figure 2A**)soft tissue mass in the left lower lobe with lobulated margins, spiculation, and pleural indentation, demonstrating increased tracer uptake (SUVmax 19.1). Multiple bilateral hilar and mediastinal lymph nodes (stations 2R, 4L, 4R, 8) also showed increased FDG uptake (SUVmax 3.9), suggestive of metastatic involvement. A bronchoscopic lung biopsy performed on July 19, 2023, confirmed poorly differentiated non-small cell carcinoma, consistent with adenocarcinoma based on immunohistochemistry: CD56(-), P40(-), NapsinA(+), CK(+), TTF-1(+), KI-67(70%+), PD-L1 (60%+, 22C3), and ALK(-). Next-generation sequencing identified an EGFR exon 21 missense mutation (c.2582T>A, p.L861Q) with an allele frequency of 13.44%, as well as PIK3CA and TP53 mutations and PD-L1 expression was 60% on tumor cells (22C3 antibody, DAKO platform) (**Figure 2B**). The patient was diagnosed with left lung adenocarcinoma, stage cT2bN2M0 IIIA (AJCC 8th edition).

Given the EGFR mutation, treatment with afatinib 30 mg daily was initiated on August 5, 2023. The patient developed hemoptysis as the main adverse event during initial therapy, which generally resolved within 3–4 days after drug discontinuation. Afatinib was rechallenged; however, follow-up chest CT on September 5, 2023(**Figure 2C**), showed disease progression with an increase in the left lower lobe lesion and enlargement of mediastinal and left hilar lymph nodes. We have discussed the diagnostic difficulty in distinguishing true radiographic progression on afatinib from pseudoprogression, given the concurrent respiratory symptom exacerbation and rising tumor markers; we clarify why progressive disease rather than pseudoprogression was concluded, leading to the switch to chemoimmunotherapy. The patient subsequently received three cycles of chemotherapy combined with immunotherapy (pemetrexed 800 mg d1+carboplatin 500 mg d1+sintilimab 200 mg d1) on September 12, October 8, November 1, 2023. A CT scan on October 31, 2023 (**Figure 2D**), after two cycles, showed a partial response (PR), which was sustained on the November 20, 2023 (**Figure 2E**) scan after three cycles. The patient tolerated chemotherapy combined with immunotherapy without significant adverse events.

On November 27, 2023, the patient underwent video-assisted thoracoscopic surgery (VATS) left lower lobectomy with lymph node dissection, achieving R0 resection. Pathological examination of the lung lesion and lymph nodes revealed no residual tumor, indicating a pathological complete response (pCR) (**Figure 2F**). Owing to the pathological complete response, the patient refused any further postoperative treatment. A follow-up chest CT on September 29, 2025 (approximately 22 months post-surgery), showed stable disease with no evidence of recurrence.

## 3 Discussion

The current treatment paradigm for resectable stage III non-small cell lung cancer (NSCLC) is increasingly centered on immunotherapy and targeted therapy. For driver gene-negative patients, regimens such as perioperative pembrolizumab plus chemotherapy—as demonstrated in the KEYNOTE-671 trial—significantly improve pathological and survival outcomes (8). However, such trials often exclude patients with driver gene alterations such as EGFR or ALK, since these tumors typically respond poorly to PD-1/PD-L1 inhibition. Relevant studies have confirmed that neoadjuvant atezolizumab is safe and effective in resectable NSCLC, with a major pathological response (MPR) rate of 20%; yet, none of the tumors harboring EGFR or ALK alterations showed radiographic response or achieved MPR (9). This poor response has been attributed to an immune “cold” tumor microenvironment, frequently characterized by the absence of concurrent PD-L1 expression and CD8+ tumor-infiltrating lymphocytes (7).However, when combined with chemotherapy, some EGFR-positive patients may still benefit. Our observations align with and extend the findings of the NEOTIDE trial, which demonstrated an MPR rate of 44.4% in EGFR-mutant NSCLC but observed no true pCR. The achievement of pCR in our two patients—both characterized by high PD-L1 expression (TPS 60%)—suggests that PD-L1 status may identify a subgroup capable of deeper pathological responses beyond MPR, a hypothesis that warrants prospective biomarker-driven validation.

Targeted therapy remains the cornerstone of treatment for EGFR-mutant non-small cell lung cancer (NSCLC): the ADAURA trial (10)stablished adjuvant osimertinib as the standard of care, and the NeoADAURA study (1) further showed that neoadjuvant osimertinib ± chemotherapy produces higher major pathologic response rates than chemotherapy alone. Nevertheless, the pathologic complete response (pCR) rate remains modest—9% with osimertinib monotherapy and only 4% when combined with chemotherapy. In contrast, immune-checkpoint inhibitor (ICI) plus chemotherapy achieves substantially higher pCR rates in EGFR/ALK-negative populations. Consistently, the phase 3 CheckMate 816 (2)and KEYNOTE-671 trials (8)demonstrated that higher PD-L1 expression (especially TPS ≥ 50%) predicts deeper pCR and greater long-term survival, forming a clear biological gradient. For resectable, locally advanced lung adenocarcinoma harboring both a sensitizing EGFR mutation and high PD-L1 (≥ 50%), no standard neoadjuvant regimen currently exists. We report two such patients (PD-L1 TPS 60%, EGFR 19-del or L861Q) who attained pCR after neoadjuvant chemotherapy plus ICI. This observation aligns with the PD-L1-driven benefit gradient described above and suggests that, in the absence of high-efficacy targeted neoadjuvant data, immunotherapy–chemotherapy combinations may represent a viable individualized strategy for this “dual-positive” subset, warranting prospective validation.

Postoperative management in our cases reflects ongoing uncertainty in clinical practice. Although Case 1 received only one cycle of adjuvant immunotherapy and Case 2 received none, both remained disease-free for over one year. Pivotal phase III trials such as Neotorch recommend completing the planned adjuvant immunotherapy course even after pCR (11). However, recent evidence indicates that postoperative adjuvant immunotherapy did not significantly improve disease-free or overall survival in patients who achieved a major pathological response (MPR), particularly pCR, following neoadjuvant chemoimmunotherapy (12). This discrepancy underscores the need for biomarker-guided individualized postoperative strategies. Recent exploratory molecular residual disease (MRD) analyses from the ADAURA trial confirmed that circulating tumor DNA (ctDNA)-based MRD detection identifies recurrence earlier than radiographic imaging, and sustained undetectable MRD during osimertinib treatment correlates strongly with long-term disease-free survival (13). Furthermore, MRD-guided treatment de-escalation has shown preliminary feasibility in advanced driver-mutated NSCLC (14). These advances suggest that for patients with a favorable pathological response and undetectable MRD, reducing treatment intensity without compromising outcomes may be feasible.

As a case report, this study is inherently limited in evidence level and cannot establish the generalizability of this treatment strategy. Nevertheless, our observations align with reports of successful conversion therapy in other driver-altered NSCLC subtypes. For example, induction therapy combining targeted agents or antibody–drug conjugates (ADCs) with immune checkpoint inhibitors has enabled surgical resection and induced profound pathological responses in patients with unresectable stage III disease harboring *BRAF* V600E (15) or *ERBB2 (*16) mutations. A common feature among these successes is that immune checkpoint inhibitors were not used alone, but rather synergized with potent tumor-killing agents—such as chemotherapy, targeted drugs, or ADCs—that may remodel the tumor immune microenvironment, potentially through mechanisms such as immunogenic cell death, thereby enabling immunotherapy to take effect.

In summary, this case report provides anecdotal evidence of pathological complete response following neoadjuvant chemoimmunotherapy in two patients with resectable stage III EGFR-mutant NSCLC and high PD-L1 expression. These preliminary observations are consistent with emerging data indicating that selected EGFR-mutant tumors may not be uniformly refractory to immunotherapy, and they raise the hypothesis that high PD-L1 expression may identify a subgroup with potentially greater sensitivity to this approach, particularly after EGFR-TKI failure. Nevertheless, given the inherent limitations of a case report—including small sample size, selection bias, and lack of a control group—these findings cannot establish efficacy or generalizability. Prospective, biomarker-driven clinical trials are required to validate this strategy. Future investigations might explore predictive biomarkers and evaluate whether pathological response assessment combined with dynamic MRD monitoring could inform personalized postoperative strategies, with the goal of maintaining survival outcomes while enabling treatment de-escalation where appropriate.

### 3.1 Patient perspective

Both patients were informed about the investigational nature of the neoadjuvant chemoimmunotherapy. Case 1 stated: “I accepted this treatment hoping for the best outcome. The dizziness after the first adjuvant cycle made me decide to stop further therapy.” Case 2 expressed: “After afatinib failed, I was worried. Achieving pCR gave me confidence, and I chose no further treatment to avoid extra side effects.

## Data Availability

The original contributions presented in the study are included in the article/supplementary material, further inquiries can be directed to the corresponding author/s.
